# Complete Genome Sequence of Lignin-Degrading *Streptomyces* sp. Strain S6, Isolated from an Oil Palm Plantation in Malaysia

**DOI:** 10.1128/MRA.01332-19

**Published:** 2020-01-30

**Authors:** Nurtasbiyah Yusof, Analhuda Abdullah Tahir, Fatimah Azizah Riyadi, Nurul Syazwani Ahmad Sabri, Fazrena Nadia Md Akhir, Nor’azizi Othman, Kah Lock Chin, Kuang Yu Tseng, Siang Tean Chin, Zuriati Zakaria, Cheng Yu Lai, Hirofumi Hara

**Affiliations:** a Department of Environmental Engineering and Green Technology, Malaysia-Japan International Institute of Technology, Universiti Teknologi Malaysia, Kuala Lumpur, Malaysia; b Department of Mechanical Precision Engineering, Malaysia-Japan International Institute of Technology, Universiti Teknologi Malaysia, Kuala Lumpur, Malaysia; c Analisa Resources (M) Sdn Bhd, Shah Alam, Selangor, Malaysia; d Inong Agriculture Co., Ltd., Taipei, Taiwan, Republic of China; e Department of Chemical Process Engineering, Malaysia-Japan International Institute of Technology, Universiti Teknologi Malaysia, Kuala Lumpur, Malaysia; University of Maryland School of Medicine

## Abstract

*Streptomyces* spp. are bacteria that are responsible for the degradation of aromatic compounds and produce secondary metabolites. Here, we present a complete genome sequence of *Streptomyces* sp. strain S6, which was isolated from an oil palm plantation, with a 7.8-Mbp liner chromosome, a GC content of 72%, and 4,266 coding sequences.

## ANNOUNCEMENT

*Streptomyces* spp. play significant roles in producing numerous extracellular enzymes to degrade macromolecules such as lignocellulose. They also produce diverse bioactive compounds, including antibiotics, which has led to the study of their genomes (1, 2). Ion Torrent sequencing was combined with Nanopore sequencing to enhance the *de novo* assembly of a complete *Streptomyces* sp. genome.

*Streptomyces* sp. strain S6, which was isolated as a lignin degrader from an oil palm plantation in Malaysia, was grown for 7 days at 30°C on W minimal medium with 10 mM Kraft lignin as the sole carbon source. Genomic DNA was extracted using the QIAamp DNA minikit (Qiagen). For the Ion X5 XL system (Thermo Fisher Scientific), a 400-bp library was constructed using the Ion Xpress Plus fragment library kit (Thermo Fisher Scientific) according to the manufacturer’s protocol. The quality of the purified library was checked using an Agilent 2100 bioanalyzer, and the library was diluted before proceeding to template preparation using the Ion Chef system (Thermo Fisher Scientific). Enriched Ion Sphere particles were loaded onto an Ion 530 chip, and sequencing was performed for 4 h. Torrent Suite software (Thermo Fisher Scientific) was used for raw data analysis, alignment, and variant calling. For MinION sequencing (Oxford Nanopore Technologies), genomic DNA was sheared using Covaris g-TUBEs and repaired with the NEBNext end repair/dA-tailing module and NEB blunt/TA ligase master mix. The library was constructed using ligation sequencing kit 1D (Oxford Nanopore Technologies) and NEB T4 DNA ligase without size selection. The purified library was loaded onto a primed MinION flow cell (FLO-MIN106) and sequenced on a GridION X5 sequencer for 48 h. Oxford Nanopore Technologies Albacore software (version 2.1.3) was used for base-calling analysis of unprocessed data.

Short reads from the Ion X5 XL system were quality trimmed using CLC Genomics Workbench software (version 11.0.1; CLC bio, Aarhus, Denmark) with the following parameters: quality score limit, 0.05; maximum number of ambiguous nucleotides, 2; and discarded reads, <400 nucleotides. The sequencing quality of long reads from the MinION sequencing was checked using FastQC version 0.11.8, and potential remaining adaptors were trimmed with Porechop. Long reads were first assembled into a draft genome using Canu version 1.8 (3). Short reads were mapped onto the long-read assembly using minimap2 version 2.16 (4) and polished with Racon version 1.3.2 (5), with the entire process being repeated 10 times. The quality of the genome in each round was assessed using CheckM version 1.0.12 (6), and the seventh round was chosen as the final version.

The total genome size is 7,875,470 bp, in a single linear chromosome, with a GC content of 72%. The accuracy of the complete genome sequence is 92.14%, and no plasmid sequence was detected using PlasmidFinder version 2.1 (7). Annotation using the NCBI Prokaryotic Genome Annotation Pipeline (PGAP) predicted 4,266 coding sequences, 18 rRNA genes, and 65 tRNAs (8). Rapid Annotations using Subsystems Technology (RAST) revealed 365 genes for the metabolism of aromatic compounds and carbohydrates that could be responsible for lignocellulose degradation (9).

### Data availability.

The complete genome sequence of *Streptomyces* sp. S6 has been deposited at DDBJ/ENA/GenBank under accession number CP040654, BioProject number PRJNA514949, and BioSample number SAMN10739602, with SRA accession numbers SRR9056892 (MinION), SRR8466423 (Ion Torrent X5 XL), and SRP180904 (fast5 files for MinION reads).
